# Progress in Medicine

**Published:** 1898-03-26

**Authors:** 


### Progress in Medicine.

### DISEASES OF THE DIGESTIVE ORGANS.

**Tumours of the Stomach.**—Schliesinger[1] draws attention to sarcomata, which generally appear late in life. The primary forms are more common than the secondary, and usually develop in the greater curvature of the stomach. Loss of weight is first noticed, and gastric disturbances follow later on, with sometimes pyloric stenosis. He noted that in seven cases H. Cl. was absent in all, and lactic acid present in five. Secondary growths may appear in the skin and spleen, but when they occur in the intestines they do not cause stricture. The usual course of the disease is from a year to a year and a half. Temporary benefit has been found from the use of arsenic.

Hayem[2] describes a variety of gastric polyadenoma which is formed on the type of Brunner's glands. There are also polypoid ones, and those formed from thickened folds of the mucous membrane. The first of the three types affects chiefly the submucous tissue, though it arises in one of the *cul de sacs* of the mucosa. It may give rise to ulceration and perforation, or even undergo a malignant change. The same author[3] discusses incomplete stenosis of the pylorus, which, as he holds, causes two types of disease. In one, the stomach is found in the morning to contain large quantities of liquid, 200 c.c. and upwards, with little or no bile, but large particles of food. Frequent vomiting takes place, and pain only occurs after food. In the other class the stomach contents are less, but bile is nearly always present, and the particles of food present are very minute. Pain is more constant, vomiting rare, and dilatation is often absent. This class he attributes to a stenosis below the pylorus, though he admits the bare possibility of continuous hyper-secretion being caused at times by spasm. Robin believes, on the other hand, that these cases are frequently due to mere spasmodic stenosis, and quotes Doyen that 46 out of 61 instances of stenosis operated on proved to be of spasmodic, and not of organic, nature. Hence he advocates a thorough trial of medical as opposed to surgical measures in these cases of continuous gastric secretion, the so-called Reichman's disease. Carle, too, in another series of operations on the stomach, several times found a permanent spasm of the pylorus without any organic disease, and thinks that while this may often be caused by an excess of acid in the stomach, the retention of food, once set up, leads to a further increase of the acid and even to a fibrous degeneration of the organ. Surgical intervention may cure these conditions by bringing about a quicker evacuation of the stomach contents, as compl tely as it relieves an organic stenosis.

McGraw, in speaking of malignant stenosis,[4] urges the ne d of earlier diagnosis of cancer. He says that if unwonted dyspepsia, which does not yield to treatment, occurs without cause in an elderly person, we should at once consider the possibility of cancer. Pain, hæmorrhage, loss of flesh, and vomiting of undigested food hours or days after eating show that stenosis of the pylorus has taken place. Finally, the operation of gastro-enterostomy is not of great danger if carried out in a person who is not exhausted by illness, and that it affords great relief when a more radical operation is not advisable. Roux,[5] also, in a discussion on continued hyper-secretion, points out that removal of the obstruction brings about amelioration, and even cures, though he does not regard the condition as due usually or wholly to organic stenosis of the pylorus. In most cases he thinks there is a special irritability of the stomach and hyper-acidity before the commencement of stasis. The latter may be due to temporary or permanent obstruction, and is accompanied by dilatation. A remarkable sequel to a malignant ulcer of the pylorus is described by Hemmeter and Ames.[6] The patient, a coloured man, suffered first from a gastric ulcer, from which a carcinoma developed with ordinary symptoms, but in the course of his disease phlegmonous gastritis appeared and pus was vomited. The post-mortem examination showed diffuse suppurative gastritis in the form of numerous miliary abscesses in the mucous and muscular coats. Death took place from perforation, and it was worth noticing that throughout the illness there was no pyrexia, and also that hydrochloric acid was present throughout in at least normal quantities, and that there was an absence of lactic acid in spite of marked stenosis and dilatation. The authors have collected reports of 56 instances of phlegmonous gastritis, and give references to all known papers on the subject.

**Dilatation.**—M. G. Boyd[7] considers that there are two forms of this disease, namely, complete and partial, the latter arising from gaseous distension, or an atonic condition of the muscular wall leading to dilatation of the cardiac end of the stomach. We meet with it in exhausting diseases, advanced phthisis, chronic alcoholism, and dyspepsias. The succussion splash seems to the author the most reliable sign of the disease in both forms, and is to be sought for an hour or two after a test meal. With this sign may be taken another when the stomach is empty, namely, a tympanitic note extending over an unusually large area. For treatment he recommends, as a temporary palliative, washing out the stomach with saline or mild disinfectant solutions, and in serious organic cases surgical measures. In minor degrees of the affection he gives drugs to prevent fermentation, alkalies before meals, occasional lavage, and liquor strychniæ.

The curious complication, tetany, which has been reported some forty times, is regarded by Gumprecht[8] and others as caused by an auto-intoxication, but there are other theories of its origin, and Gumprecht himself, after examining the stomach contents and urine, could find no poison at the time of the tetany which could be looked upon as the cause of an auto-intoxication. Berlitzheimer[9] also failed to discover any ptomaine in the stomach contents. Gastroptosis has been referred to numerous causes, but Kuttner and Dyer[10] consider with some reason that tightlacing and pregnancy act less by any direct effect than by interference with the motor functions of the stomach, which produces atony and dilatation. Symptoms are often absent, but motor power is deficient with subacidity in early stages. They recommend abdominal binders, regulation of the bowels, massage, electricity, and the administration of strychnia. It may be necessary to correct displacement of other abdominal organs by operative measures. W. Carr[11] describes an instance of dilatation complicated with peripheral neuritis. There was partial but symmetrical loss of motion and sensation, tenderness of the muscles, loss of knee-jerks, without girdle pains or affection of the sphincters or pupils, and fairly complete recovery when the dilatation was treated in the usual way. No other cause for the neuritis could be found, and the author regards the affection as caused by the absorption of some toxin from the dilated stomach, though similar cases do not seem to have been recorded. The dilation had existed probably for some twelve years, and more marked effects may thus have been caused than in ordinary cases of shorter duration. Indeed, Dr. Henry Waldo has given an account of a case of peripheral neuritis following apparently the ingestion of poisonous food. We may here refer to another complication described by H. D. Rolleston.[12] A stenosis of the pylorus, caused by a chronic ulcer, was accompanied not only by dilatation, but also by so much inflammation that the head of the pancreas and the bile duct were involved, and obstructive jaundice was set up. The presence of a hard mass, together with jaundice, led to a diagnosis of malignant disease of the pylorus, which was disproved by the post-mortem examination. There had been no signs of gastric ulcer, and the remarkable extension of the peripyloric fibrosis, which caused enlargement of the head of the pancreas so great as to simulate carcinoma, and actually to obstruct the common duct, is apparently unprecedented. A similar change in the head of the pancreas may be produced when a gall-stone is lodged in the common duct, and it is pointed out that an operator may be thus misled, and mistake it for a malignant growth.

**Ulcer.**—A careful analysis of symptoms and condition of perforated gastric ulcer is given by Walter Broad bent.[13] The statistics at St. Mary's Hospital show that perforation occurred in 6 per cent. of the cases of ulcer, though Willink gives only 3 per cent. As Brinton laid down, perforations in the anterior surface are the most frequent, the lesser curvature coming next, while the posterior surface is rarely affected. In nearly half the cases general peritonitis does not take place, especially when the peforation is near the lesser curvature. If more than 1½ in. from this, and on the anterior surface, general peritonitis is, however, almost certain. Premonitory symptoms are scanty; even those of gastric ulcer are sometimes absent before the sudden onset of peritonitis, but occasionally attacks of violent pain occur a few days before perforation, and are due to slight peritonitis round the base of the ulcer. When perforation has taken place, symptoms of general peritonitis may come on; or if a subphrenic abscess be formed, tympanitic resonance on the left side may rise as high as the fifth space, while the liver dulness in the nipple line may remain normal. The patient in this case does not become rapidly worse as in general peritonitis. Vomiting ceases in two or three days, the abscess increases in size, and the heart is pushed upwards and outwards, and the left lung is compressed. On the other hand, vomiting of pus and lymph is a marked feature after the first day, when rupture occurs into the lesser omental cavity. A tumour is soon felt in the epigastric region, and a subphrenic abscess may develop later on. There may in some cases be so few initial symptoms that the diagnosis from pleurisy, empyæma and pneumothorax, and even pericarditis or biliary colic, may be difficult.

**Gastric Crises.**—Œttinger[14] discusses certain attacks of absolute intolerance of food, lasting a few hours or days, appearing at intervals in persons who enjoy thorough health. There is often violent pain, which led Charcot to look on them as symptoms of tabes, but they occur in persons either in good health or suffering from hysteria or other forms of nerve disease. Unlike ordinary hysterical vomiting, the attacks last only for a short time; there is hyper-secretion of gastric juice in many cases. The author looks on them as a disease of the gastric nerves, and probably the same as the intermittent type of Reichman's disease. The three cardinal symptoms are pain, vomiting, and alteration of the gastric secretion coming on at intervals, often in the absence of any other disease.

Buzdygan[15] has investigated the effect of the administration of iron in mild preparations on the gastric secretion. He finds that where the secretion is normal iron has no effect whatever, but where there is hyper-acidity iron increases the derangement and the pains after food which such patients complain of. On the other hand, where there is a deficiency of acid, iron acts as a stimulant and increases the amount, causing improved digestion and removing pain after food.

[1] Amer. J. Med. Sc., Sept. [2] B.M.J., Dec. 18. [3] Int. Med. Mag., Oct. [4] Amer. Med. Surg. Bulletin, Oct. 25. [5] Med. Rec., Sept. 4. [6] Med. Rec., Sept. 18. [7] Dublin J. Med. Sc., Dec. [8] Amer. J. Med. Sc., Nov. [9] Amer. J. Med. Sc., Dec. [10] Int. Med. Mag., Oct. [11] Lancet, Sept. 18. [12] Practit., Nov. [13] B.M.J., Oct. 30. [14] B.M.J., Sept. 30. [15] Les Nouv. Remèdes, Oct. 26.

